# Specific Features of Patients Under 40 Years Old With Small-to-Medium-Sized Arterial Deterioration

**DOI:** 10.3389/fsurg.2022.808383

**Published:** 2022-02-24

**Authors:** Kazuyoshi Matsubara, Natsumi Fukuhara, Katsuyuki Hoshina, Kazuhiro Miyahara, Masamitsu Suhara, Ryosuke Taniguchi, Mitsuru Matsukura, Toshio Takayama

**Affiliations:** Division of Vascular Surgery, Department of Surgery, Graduate School of Medicine, The University of Tokyo, Tokyo, Japan

**Keywords:** arterial abnormalities, Behçet's disease, Ehlers-Danlos syndrome, multiple lesions, polysurgery

## Abstract

**Background:**

Arterial deterioration is mostly caused by atherosclerosis, which progresses with age. However, we have observed serious backgrounds or etiologies in younger patients with non-atherosclerotic diseases and deterioration of small-to-medium-sized arterial lesions. Therefore, we aimed to identify the specific features of patients aged <40 years with deterioration of small-to-medium-sized arteries.

**Methods:**

We selected patients who were admitted to our department from 1995 to 2019 with deterioration of small-to-medium-sized arteries (aneurysms, dissection, rupture, or arterial injury/damage) and focused on the cohort aged <40 years. We examined the backgrounds or etiologies of the patients including genetic and inflammatory diseases, which might have caused the arterial deterioration.

**Results:**

Consequently, more than half (54.1%) of the patients aged <40 years had non-atherosclerotic comorbid diseases. However, the number of deteriorated arterial lesions was higher in patients aged <40 years than in patients aged ≥40 years (3.13 vs. 1.33 lesion/patient; *P* = 0.011). Furthermore, the data analysis of patients with multiple arterial lesions (≥3) revealed that the younger population tended to have more specific backgrounds or etiologies, notably Ehlers-Danlos syndrome and Behçet's disease. There were no differences in the all-cause mortality and cardiovascular disease-related mortality between patients aged <40 and ≥40 years (*P* = 0.89 and 0.29, respectively).

**Conclusions:**

Over 50% of patients aged <40 years with deterioration of small-to-medium-sized arteries had non-atherosclerotic, specific clinical backgrounds or etiologies, including genetic and inflammatory diseases. In addition, they exhibited more arterial lesions than older patients.

## Introduction

Arterial deterioration can cause catastrophic events, such as rupture or hemorrhage, which sometimes require immediate surgical intervention. In older patients, atherosclerosis causes arterial wall deterioration accompanied by various clinical features, such as calcification, plaque, tortuosity, and elongation. Infections that can cause aneurysms or arterial dissections can be detected clinically by the resultant elevated levels of inflammatory markers or blood cultures. Patients that present without such obvious symptoms are considered to likely have specific backgrounds or etiologies, including genetic and inflammatory diseases. Young patients with arterial deterioration are initially suspected of having genetic diseases, such as Marfan syndrome (MS), Loeys-Dietz syndrome (LDS), and Ehlers-Danlos syndrome (EDS), which are well-known hereditary connective tissue disorders that cause vascular abnormalities, which can result in aneurysms and dissection (1). Behçet's disease (BD) and Takayasu's disease are two major inflammatory diseases that are relatively common in Asia; that cause graft occlusion, aneurysms at different sites, and anastomotic aneurysms in a short period (2). Furthermore, segmental arterial mediolysis (SAM), an uncommon, non-atherosclerotic, non-inflammatory arteriopathy, has a mortality rate of ~50% and requires urgent intervention in cases of acute splanchnic aneurysm rupture; therefore, it should also be included in the differential diagnosis for arterial deterioration (3).

Recognizing these diseases and their clinical features is critical for selecting the appropriate surgical treatment, making decisions about medication, and devising follow-up plans, especially for younger patients. Thus, we aimed to identify the specific features of patients aged <40 years with deterioration of small-to-medium-sized arteries. We selected the small-to-medium-sized arterial lesions excluding the aorta and the iliac arteries to eliminate the atherosclerotic etiology especially in the elderly patients. For this study, we selected patients with deteriorating arterial lesions who had undergone surgery in our hospital. We focused on patients aged <40 years, whose arteries are non-atherosclerotic, and on multiplicity of lesions that could be associated with tissue fragility or a tendency toward arterial destruction.

## Materials and Methods

We performed a search of the medical records from 1995 to 2019 in the Department of Vascular Surgery at the University of Tokyo Hospital, a tertiary referral hospital in Tokyo, using the following search terms: “aneurysm,” “dissection,” “rupture,” “anastomosis/anastomotic,” “injury,” and “damage,” while excluding the terms, “aorta/aortic,” “iliac,” “iatrogenic,” “infection/infectious,” and “trauma/traumatic.” Thereafter, we selected patients who were admitted to our department with deteriorating arterial lesions (except aortic and iliac artery lesions). We then examined the following possible relevant backgrounds or etiologies as possible causes of arterial deterioration: genetic diseases, including MS, LDS, EDS, von Recklinghausen's disease, and Osler-Weber-Rendu disease; vasculitis, including BD, Takayasu's vasculitis, and Churg-Strauss syndrome; and some rare etiologies, such as median arcuate ligament compression syndrome (MALS). MALS and subsequent celiac artery stenosis, which have been reported to be associated with aneurysm formation in the pancreaticoduodenal arterial arcade, were considered as part of the etiologies (4, 5). SAM and fibromuscular dysplasia (FMD) were also included as important backgrounds that cause arterial deterioration. We excluded pseudoaneurysms after pancreatitis or infection, not caused by arterial fragility.

Patients were classified according to age (<40, 40–49, 50–59, 60–69, 70–79, and ≥ 80 years), number of deteriorating arterial lesions, multiple lesions (≥ 3), and comorbidities, including hypertension, diabetes mellitus, hyperlipidemia, current smoking, obesity (body mass index ≥25), chronic obstructive pulmonary disease, and renal dysfunction (estimated glomerular filtration rate <45 mL/min/1.73 m^2^). The differences in mortality, number of lesions, and number of patients with multiple lesions were analyzed between the <40-year age group and the other age groups. The numbers of arterial lesions were counted independently.

The categorical variables were presented as numbers and percentages, and the Wilcoxon rank-sum test was performed for continuous variables. Freedom from all-cause mortality and cardiovascular disease-related mortality rate for 10 years were analyzed with the Kaplan–Meier method using JMP® Pro, version 16 (SAS Institute Inc., Cary, NC, 1989–2021) and sample script (downloaded from https://www.jmp.com/japan/support/faq/stat_03.shtml). Statistical significance was set at *P* < 0.05.

The use of the medical records for this study was approved by the Ethics Committee of the University of Tokyo Hospital (Approval no. 3316-3).

## Results

**Figure 1:**
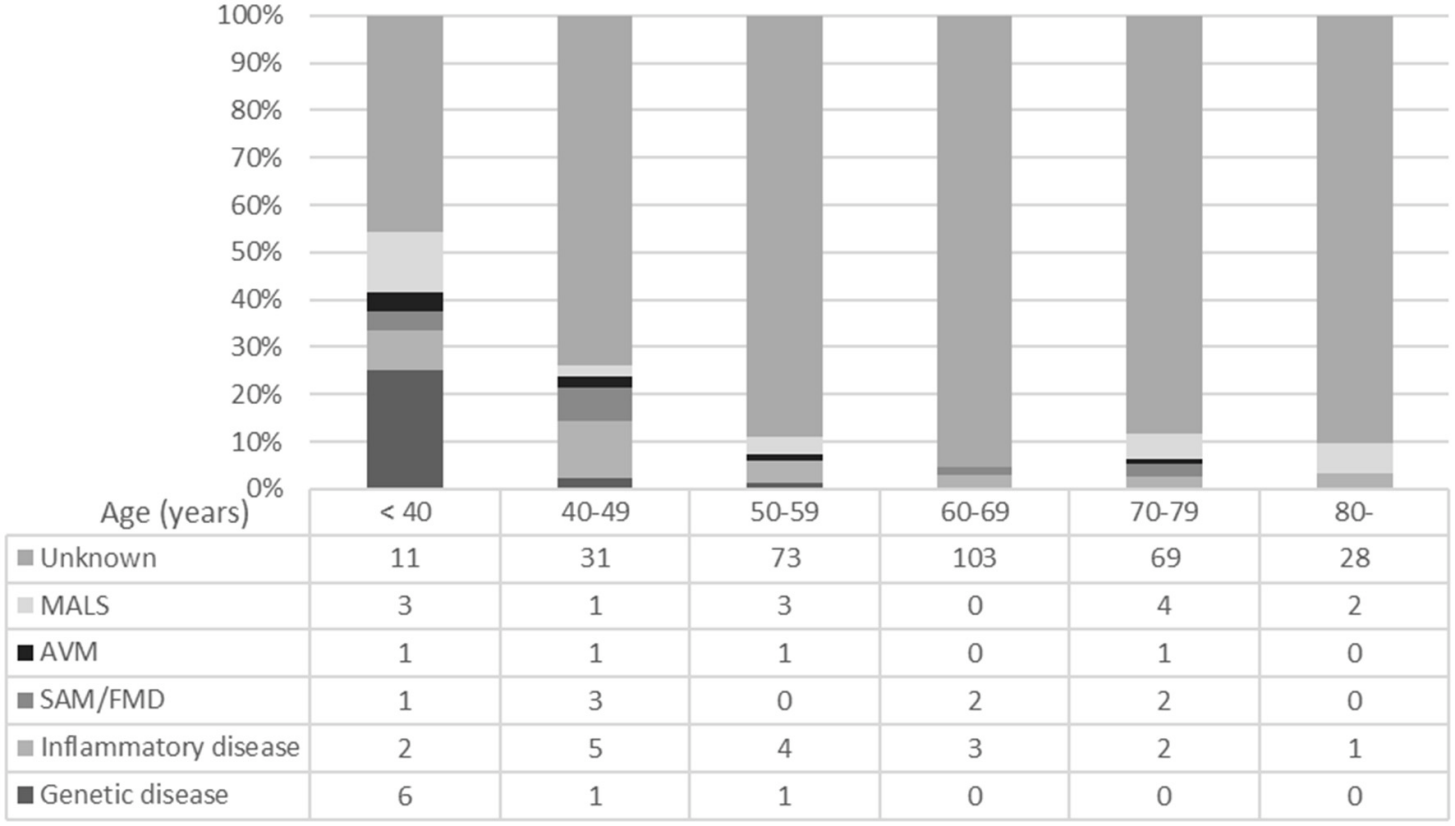
Backgrounds or etiologies of each age group. MALS, median arcuate ligament compression syndrome; AVM, arteriovenous malformation; SAM, segmental arterial mediolysis; FMD, fibromuscular dysplasia.

A total of 364 patients with 524 lesions were selected. There were 75 lesions in 24 patients in the <40-year age group (Table 1). The age of the patients at the time of the first admission to our hospital ranged from 11 to 39 years, with a mean ± standard deviation of 29.4 ± 7.2 years. The proportion of patients with backgrounds or etiologies possibly relevant to arterial deterioration was high (54.1%) in the <40-year age group (Figure 1).

**Table 1:** Characteristics of the study population.

**Age (year-old)**	**<40**	**40–49**	**50–59**	**60–69**	**70–79**	**80**~****
Number of patients	24	42	82	107	78	31
Number of lesions	75	69	110	138	94	41
(Lesions/a patient)	3.13	1.33				
Patients with multiple lesions ≥3	7	7	6	9	3	2
	29%	7.9%				
<**Morbidities**>						
Hypertension	3 (14%)	14 (36%)	34 (55%)	50 (60%)	43 (68%)	19 (63%)
Diabetes mellitus	1 (4%)	0 (0%)	8 (12%)	16 (19%)	13 (19%)	19 (63%)
Dyslipidemia	1 (4%)	12 (31%)	23 (34%)	33 (38%)	21 (30%)	19 (63%)
Smoking	8 (38%)	20 (51%)	26 (44%)	40 (51%)	15 (25%)	19 (63%)
Obesity (BMI≥25)	2 (10%)	10 (31%)	17 (40%)	15 (22%)	12 (25%)	10 (33%)
COPD	0 (0%)	2 (9%)	6 (16%)	12 (21%)	11 (26%)	5 (24%)
Renal dysfunction (eGFR < 45)	0 (0%)	0 (0%)	3 (4%)	0 (0%)	6 (8%)	11 (37%)

*BMI, Body Mass Index; COPD, chronic obstructive pulmonary disease; eGFR, estimated glomerular filtration rate*.

The number of deteriorated arterial lesions was greater in the <40-year age group than in the ≥40-year age group (3.13 vs. 1.33 lesion/patient; *P* = 0.011). Similarly, the percentage of patients with multiple arterial lesions (≥3) was higher in the <40-year age group than in the ≥40-year age group (7 [29%] vs. 25 patients [7.9%]; *P* = 0.0036) (Table 1 and Figure 1). Older patients had more comorbid conditions; however, no specific trends were identified (Table 1).

**Figure 2:**
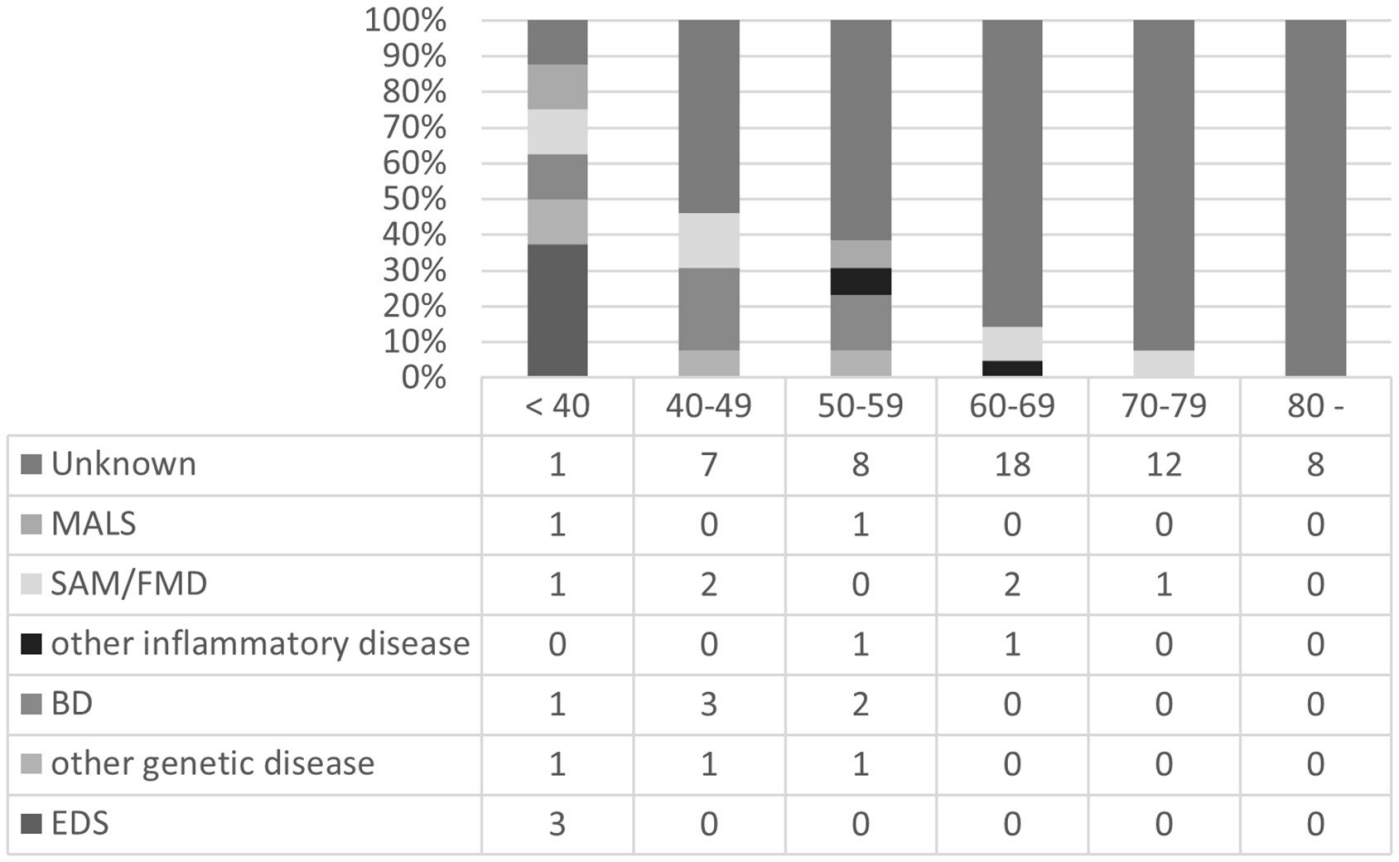
Backgrounds or etiologies of patients with multiple arterial lesions (≥3) in each age group. MALS, median arcuate ligament compression syndrome; SAM, segmental arterial mediolysis; FMD, fibromuscular dysplasia; BD, Behçet's disease; EDS, Ehlers-Danlos syndrome.

The profiles of patients with multiple arterial lesions (≥3) were compared (Figure 2). In the <40-year age group, 8 out of 9 patients (89%) had possible causative backgrounds or etiologies of arterial deterioration, which indicated the significance of these factors in younger patients with multiple lesions. Among them, EDS and BD were notable in younger patients [3 cases of EDS, 1 case of LDS, and 1 case of BD in the <40-year age group (56%) and 3 cases of BD in the 40–49-year age group (23%)].

The distribution of the lesions is compared in Table 2. The percentage of the lower limb artery deterioration, which is often associated with arteriosclerosis in the <40-year age group (5%) was smaller than that in the ≥40-year age group (21%).

**Table 2:** Comparison of the lesions between the two groups.

**Age (year-old) Lesion**	**Under 40 (75 lesions)**	**40 or over (452 lesions)**
Splenic artery	11 (15%)	114 (25%)
Renal artery	11 (15%)	93 (21%)
Hepatic artery	2 (3%)	26 (6%)
GDA/PDA	4 (5%)	26 (6%)
Mesenteric/colic artery	7 (9%)	18 (4%)
Celiac artery	1 (1%)	21 (5%)
Gastric/gastroepiploic artery	3 (4%)	7 (2%)
Carotid artery	13 (17%)	18 (4%)
Lower limb artery	4 (5%)	95 (21%)
Upper limb artery	4 (5%)	3 (0.7%)
Others	15 (20%)	31 (7%)

**Figure 3:**
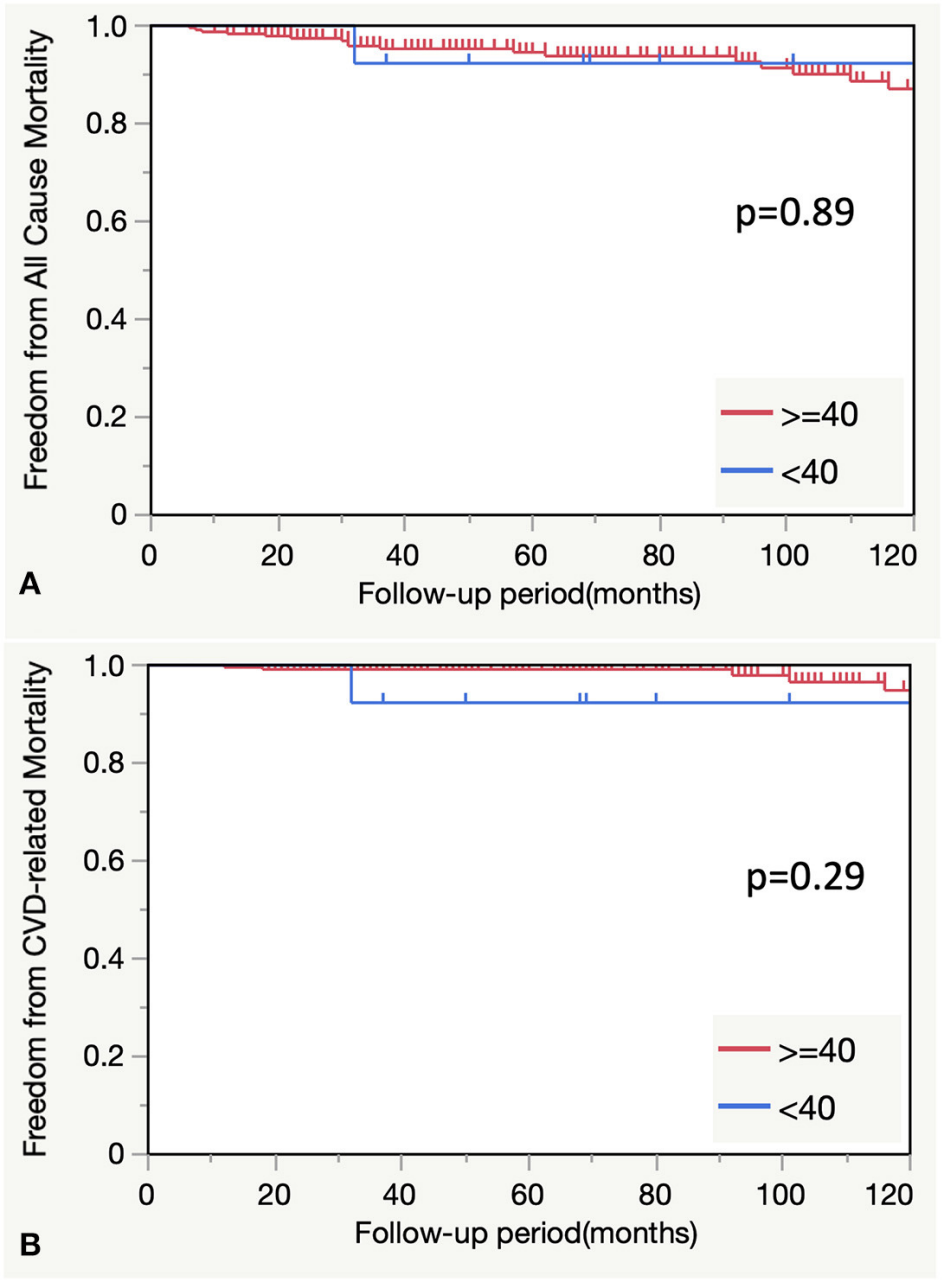
**(A)** Comparison of all-cause mortality between the <40-and ≥40-year age groups. **(B)** Comparison of CVD-related mortality between the <40- and ≥40-year groups. CVD, cardiovascular disease.

There were no statistically significant differences in the freedom from all-cause and cardiovascular disease-related mortality between the <40-year age group and older age groups (*P* = 0.89 and 0.29, respectively) (Figure 3).

## Discussion

In this study, we identified the specific features of patients aged <40 years with deterioration of small-to-medium-sized arteries. We observed that more than half of the patients aged <40 years with deterioration of small-to-medium-sized arteries had associated clinical backgrounds or etiologies, including genetic and inflammatory diseases. In addition, they had more lesions compared to older patients. In patients with multiple lesions, younger age was positively associated with backgrounds or etiologies related to arterial deterioration.

When operating on patients with arterial deterioration, the genetic background should be considered because some genetic diseases result in extremely fragile tissues, which may be affected during surgery. Such patients are susceptible to recurrent adverse events after the operation, which leads to a low life expectancy (1, 6). LDS, caused by an autosomal dominant mutation in the genes coding for the transforming growth factor-beta receptor, and vascular EDS, caused by mutations in the collagen type III alpha 1 gene, have poor prognoses (6). The average life expectancies of patients with LDS and EDS were reported to be 37 and 51 years, respectively (6–8). Early diagnosis is important, especially, for surgeons, and surgical techniques such as felt reinforcement of the vessel wall and placement of a pledget buttress on the puncture site of the access vessel should be considered intraoperatively (9). In addition, careful radiological follow-up and genetic counseling should be provided postoperatively.

Although new-generation sequencing technologies have drastically improved the ability to identify unknown genes, many clinicians in Japan do not know how to access the genetic diagnostic system. The Initiative on Rare and Undiagnosed Disease (IRUD) was launched in 2015 with the aim of constructing a comprehensive medical network and an international data-sharing framework. The criteria for referral to the IRUD centers are as follows: the patient remains undiagnosed for ≥6 months, symptoms affect their daily lives and objective signs are not confined to a single organ or there is direct or indirect evidence of a likely genetic etiology (10). Most of the young patients in this study met these criteria. Hence, we assume that with further genetic examination, more potential patients with genetic disorders might be detected in our cohort based on the IRUD criteria.

Vasculitis should be ruled out when arterial deterioration is diagnosed. BD, Takayasu's aortitis, and polyarteritis nodosa have their diagnostic criteria and unique clinical features. Although it is difficult to differentiate between these diseases based only on the site of the arterial lesions as the interval between the occurrence of arterial lesions may provide a clue. For instance, the interval between the time of operation and the diagnosis of an anastomotic false aneurysm has been reported to be shorter in patients with BD (2) than in patients with Takayasu's disease (11) and atherosclerosis (12). The short intervals and multiple lesions present in BD and EDS patients may indicate the severity of the disease.

FMD is a non-atherosclerotic and non-inflammatory segmental involvement of the musculature of the arterial wall, which is sometimes likened to SAM. Multiple aneurysms are a common feature of these diseases; however, FMD is seen in middle-aged women and is usually asymptomatic with a lower risk of rupture. The most common sites of FMD are the renal and carotid arteries, rather than the visceral arteries that are commonly affected in patients with SAM (2, 13). A bead-like appearance and acute remodeling after hemodynamic changes due to endovascular treatments were observed in cases of SAM but rarely in other diseases (14, 15). Although a definite diagnosis with pathological examination is sometimes impossible in this era of endovascular surgeries, a drastic change in arterial morphology after vascular intervention is one of the characteristic features (15).

There was no difference in freedom from all-cause and cardiovascular-related mortalities between patients aged <40 years and those aged ≥40 years in this study. However, patients aged <40 years demonstrated more specific backgrounds or etiologies and multiple lesions which may have been the reasons for the low mortality rate in this group. We assume that these two strategies were beneficial to this group: timely reintervention through careful follow-up plans with short intervals and maintenance of inflammation remission in cases of BD or Takayasu's disease using steroids or immunosuppressants.

A special case we considered in this study was that of a patient aged <40 years who presented with multiple arterial lesions and an unknown etiology. He had a history of hemorrhage of the internal thoracic artery and was referred to our hospital with a diagnosis of ruptured iliac artery aneurysm. Further investigations revealed dissection of the celiac, common hepatic, and splenic arteries. The multiplicity of arterial deterioration and variety of clinical features were indicative of a genetic or vasculitis element. However, genetic studies and immunological examinations did not reveal any background diseases. Similarly, some younger patients in our study cohort had an unknown etiology, which could not be analyzed in the current survey. Treatment options, such as endovascular treatment, open surgery, or observation, for such patients should be selected by considering the patients' condition, inflammation status, interval of recurrence, and condition of the deteriorating lesions.

This study had some limitations. First, this was a retrospective, observational study with a small sample size in one department. Second, background data was not available for this retrospective analysis in cases of rupture leading to death. Third, the genetic survey did not fully cover all the patients. Fourth, the surveillance of vasculitis with immunological studies was incomplete in patients who underwent surgery in the past. Further studies considering larger populations and with easier access to genetic diagnostic systems, such as IRUD, will be necessary to establish algorithms for the differential diagnosis of diseases underlying arterial deterioration.

## Conclusions

More than half of patients aged <40 years with deterioration of small-to-medium-sized arteries had non-atherosclerotic, specific clinical backgrounds or etiologies, including genetic and inflammatory diseases. They also had more lesions compared to older patients. In patients with multiple lesions, backgrounds or etiologies associated with arterial deterioration were strongly related to younger age.

## Data Availability Statement

The raw data supporting the conclusions of this article will be made available by the authors, without undue reservation.

## Ethics Statement

The studies involving human participants were reviewed and approved by the Ethics Committee of the University of Tokyo Hospital (Approval no. 3316-3). Written informed consent to participate in this study was provided by the participants' legal guardian/next of kin.

## Author Contributions

KMa, NF, and KH: conception and design of the work. KH, KMi, MS, RT, MM, and TT: acquisition of data. KMa and NF: analysis or interpretation of data for the work. KH: investigation. All authors: critical review and revision, final approval of the article, and accountability for all aspects of the work.

## Conflict of Interest

The authors declare that the research was conducted in the absence of any commercial or financial relationships that could be construed as a potential conflict of interest.

## Publisher's Note

All claims expressed in this article are solely those of the authors and do not necessarily represent those of their affiliated organizations, or those of the publisher, the editors and the reviewers. Any product that may be evaluated in this article, or claim that may be made by its manufacturer, is not guaranteed or endorsed by the publisher.

## References

[1] Meester JAN Verstraeten A Schepers D Alaerts M Laer LV Loeys BL. Differences in manifestations of Marfan syndrome, Ehlers-Danlos syndrome, and Loeys-Dietz syndrome. Ann Cardiothorac Surg. (2017) 6:582–94. 10.21037/acs.2017.11.03 29270370 PMC5721110

[2] Hosaka A Miyata T Shigematsu H Shigematsu K Okamoto H Ishii S. Long-term outcome after surgical treatment of arterial lesions in Behçet's disease. J Vasc Surg. (2005) 42:116–21. 10.1016/j.jvs.2005.03.019 16012460

[3] Pillai AK Iqbal SI Liu RW Rachamreddy N Kalva SP. Segmental arterial mediolysis. Cardiovasc Intervent Radiol. (2014) 37:604–12. 10.1007/s00270-014-0859-4 24554198

[4] de Perrot M Berney T Deleaval J Buhler L Mentha G Morel P. Management of true aneurysms of the pancreaticoduodenal arteries. Ann Surg. (1999) 229:416–20. 10.1097/00000658-199903000-00016 10077055 PMC1191708

[5] Miyahara K Hoshina K Nitta J Kimura M Yamamoto S Ohshima M. Hemodynamic simulation of pancreaticoduodenal artery aneurysm formation using an electronic circuit model and a case series analysis. Ann Vasc Dis. (2019) 12:176–81. 10.3400/avd.oa.19-00005 31275470 PMC6600102

[6] Loeys BL Schwarze U Holm T Callewaert BL Thomas GH Pannu H. Aneurysm syndromes caused by mutations in the TGF-beta receptor. N Engl J Med. (2006) 355:788–98. 10.1056/NEJMoa055695 16928994

[7] Pepin M Schwarze U Superti-Furga A Byers PH. Clinical and genetic features of Ehlers-Danlos syndrome type IV, the vascular type. N Engl J Med. (2000) 342:673–80. 10.1056/NEJM200003093421001 10706896

[8] Frank M Adham S Seigle S Legrand A Mirault T Henneton P. Vascular Ehlers-Danlos Syndrome: long-term observational study. J Am Coll Cardiol. (2019) 73:1948–57. 10.1016/j.jacc.2019.01.058 30999998

[9] Brooke BS Arnaoutakis G McDonnell NB Black JH. Contemporary management of vascular complications associated with Ehlers-Danlos syndrome. J Vasc Surg. (2010) 51:131–9. 10.1016/j.jvs.2009.08.019 19879095 PMC6042287

[10] Adachi T Kawamura K Furusawa Y Nishizaki Y Imanishi N Umehara S. Japan's initiative on rare and undiagnosed diseases (IRUD): towards an end to the diagnostic odyssey. Eur J Hum Genet. (2017) 25:1025–28. 10.1038/ejhg.2017.106 28794428 PMC5558173

[11] Miyata T Sato O Deguchi J Kimura H Namba T Kondo K. Anastomotic aneurysms after surgical treatment of Takayasu's arteritis: a 40-year experience. J Vasc Surg. (1998) 27:438–45. 10.1016/S0741-5214(98)70318-0 9546229

[12] McCabe CJ Moncure AC Malt RA. Host-artery weakness in the etiology of femoral anastomotic false aneurysms. Surgery. (1984) 95:150–3. 6695332

[13] Sanches-Baya M Ramos Galí A Barros-Membrilla A Guerrero R Villalba J Martinez MJ. Renal artery dissection in a young woman: diagnoses beyond fibromuscular dysplasia. Nephron. (2019) 143:128–32. 10.1159/000501039 31394546

[14] Hashimoto T Deguchi J Endo H Miyata T. Successful treatment tailored to each splanchnic arterial lesion due to segmental arterial mediolysis (SAM): report of a case. J Vasc Surg. (2008) 48:1338–41. 10.1016/j.jvs.2008.05.056 18971044

[15] Nishikawa Y Hoshina K Sasaki H Hosaka A Yamamoto K Okamoto H. Acute remodeling of an adjoining aneurysm after endovascular treatment of a ruptured splanchnic arterial aneurysm: A case of clinically diagnosed segmental arterial mediolysis. Ann Vasc Dis. (2012) 5:449–53. 10.3400/avd.cr.12.00060 23641269 PMC3641545

